# Effects of Sleep Deprivation on Surgeons Dexterity

**DOI:** 10.3389/fneur.2019.00595

**Published:** 2019-06-11

**Authors:** Tommaso Banfi, Erika Coletto, Paola d'Ascanio, Paolo Dario, Arianna Menciassi, Ugo Faraguna, Gastone Ciuti

**Affiliations:** ^1^The BioRobotics Institute, Scuola Superiore Sant'Anna, Pontedera, Italy; ^2^Norwich Research Park Innovation Centre, Quadram Institute of Bioscience, Norwich, United Kingdom; ^3^Department of Translational Research and New Technologies in Medicine and Surgery, University of Pisa, Pisa, Italy; ^4^Department of Developmental Neuroscience, IRCCS Fondazione Stella Maris, Pisa, Italy

**Keywords:** surgeon, sleep deprivation, dexterity, surgery, surgeon performance, review

## Abstract

Sleep deprivation is an ordinary aspect in the global society and its prevalence is increasing. Chronic and acute sleep deprivation have been linked to diabetes and heart diseases as well as depression and enhanced impulsive behaviors. Surgeons are often exposed to long hour on call and few hours of sleep in the previous days. Nevertheless, few studies have focused their attention on the effects of sleep deprivation on surgeons and more specifically on the effects of sleep deprivation on surgical dexterity, often relying on virtual surgical simulators. A better understanding of the consequences of sleep loss on the key surgical skill of dexterity can shed light on the possible risks associated to a sleepy surgeon. In this paper, the authors aim to provide a comprehensive review of the relationship between sleep deprivation and surgical dexterity.

## Introduction

Sleep deprivation is defined as “Abnormal sleep that can be described in measures of deficient sleep quantity, structure and/or sleep quality” (1). A wide body of literature supports the evidence that daily sleep duration plays an essential role in maintaining general healthy functioning. Moreover, sleep deprivation (SD) prevalence is increasing in the society (2), creating a raising challenge in managing daily performance deficits due to sleep loss. The detrimental effects associated to SD are of particular importance in specific populations as a cause of impaired performance, implying dangerous to deadly consequences (3). In parallel to the widely studied military population, surgeons are traditionally exposed to considerable levels of acute total and chronic SD (3–5), causing significant concerns on safety issues associated with sleepy surgeons (6, 7).

To decrease the chance for a work-related accident to occur, it is necessary to prevent excessive sleepiness during work hours. It has been shown, in fact, that having sleep restricted to 6 h per day for an entire week leads to the same neurobehavioral performances observed in subjects sleep deprived for the whole night (8). This impairment is also comparable to the one recorded in someone considered alcohol intoxicated under the law limits permission (9).

Given the relatively broad spectrum of physiological sleep duration in humans, there is no consensus in advising about a specific recommended sleep duration. Individuals do not show common SD related deficits when sleeping at least 8 h per day (8). Therefore, a similar amount of sleep is also recommended by the national sleep foundation guidelines for adults subjects (10).

In the medical context, interns working more than 80 h per week have a significantly higher number of attentional deficits during overnight shifts (11). Limitations on work hours are varies across countries in the world. The European working time directive regulates maximum work hours in Europe for all workers. The maximum number of consecutive hours allowed is 13 and weekly average duty hours are limited to 48 (12). In the United States of America, the Accreditation Council for Graduate Medical Education (ACGME) regulates physicians in training duty hours. Thirty hours is the current limit of consecutive hours on duty, moreover a weekly work hours limitation is set to 80 h (13). In New Zealand physicians in training should not work more than 16 consecutive hours, weekly work hour limit is set to 72 h (14). Taking a broader perspective, it is worth mentioning that a marked reduction in average work hours in industrialized countries was observed in the last century (15).

The number of serious medical errors in intensive care unit were also found to be higher when a work schedule including long night shifts (24 h or more) were adopted (4). Interns are at higher risk of percutaneous injuries after a night shift or during night time (16). The risk of incurring in a motor vehicle accident in the next month was found to be raised by 9.1% (95% CI, 3.4–14.7%) for each scheduled 24-h shift during that month (17). Alertness, measured objectively using the Psychomotor Vigilance Task (PVT), a standardized task based on the measure of reaction times (18, 19), was significantly worse in interns (although pertaining to an internal medicine department and not to a surgical one) after on-call shifts when compared to regular shifts (20). Heavy call rotations (calls occurring every fourth or fifth night) induced neurobehavioral deficit that were comparable to those related to a Blood Alcohol Concentration (BAC) of 0.04 to 0.05%, inters had 40% more attentional lapses and their subjective ability to evaluate their impairment level appeared to be limited and non-uniform across tasks (21). Activities occurring in the day after an on-call night could also be somewhat altered by the residual effects of SD. In this situation a 24% reduction of correct adenoma detection during colonoscopies, performed by experienced surgeons, were found (22). During on-call night shifts interns slept on average 6.93 h (6.84–7.03 h; 95% CI), significantly less than the average 7.18 h (7.06–7.30 h; 95% CI) recorded in residents who did not undergo shifts. Moreover in 17.5% of the nights residents did not sleep at all. Residents were shown to present an amplified negative emotional response to disruptive events as well as reduced positive effects of goal-enhancing ones (23). A nationwide survey study conducted among 2,737 American residents of various specialties showed that extended duration shifts, and their frequency, raised the risk of self-reported medical errors, patient fatalities, and attentional failures (3). In the same study, it was reported that the chance of incurring in attentional failures within the medical settings (e.g., falling asleep during patient examination or surgical procedure) was significantly higher for residents that followed a working schedule that included long shifts. It is important to note that self-reported medical errors are known to significantly worsen physicians' quality of life and also that they enhance burnout feelings (24). Associated to these consequences were a decreased empathy toward patients and an increase in the probability of incurring in self-perceived medical errors, hence building a negative spiraling toward, again, more perceived errors and higher distress.

Although the detrimental effects associated to SD have been widely investigated, only few studies have explored the effects of SD on surgical performance and the review of the conclusions of those papers lead to little or inconclusive evidences (25–30).

This review aims to list and analyse the most relevant papers about SD and its effects in surgeons' dexterity and abilities, pointing out the differences of the findings and paradigms of investigation. Moreover, hereby we discuss methodological issues in the systematic review of the relevant studies, as well as practical suggestions to surgeons and health organizations.

The first part of the paper reviews the current literature and the state of the art about the effect of SD on surgical dexterity. Then, we will move on discussing the effects of psychostimulants on sleep deprived surgeons. Finally, we will review current safe countermeasures that could be adopted in the attempt to counteract the detrimental effects of SD on the work place.

A comprehensive search of PubMed until December 2018 was performed. Only papers written in English were included. The search query selected was: Sleep AND deprivation OR restriction OR fatigue AND surgeon^*^ which produced 1,802 results.

Included study type were: previous systematic reviews, experimental studies comparing direct measures of surgical performance obtained using previously validated surgical rating scales or assessment methods (e.g., laparoscopic simulators), randomized controlled trials, non-randomized studies comparing groups' or subjects' performances. Studies administering experimental modifications that included exposure of subject to acute sleep deprivation or chronic sleep restriction were included in the review.

Papers lacking direct measures of sleep in either a subjective or objective way or concerning purely the study of surgeons physical or neuromuscular fatigue were excluded. Studies regarding retrospective analysis of surgical outcome after sleep deprivation or restriction were also excluded. Finally, communications to national or international meetings were excluded in the review.

After application of study inclusion and exclusion criteria, we performed an analysis of the references reported in included studies to identify further relevant papers following previously defined criteria. Therefore, starting from 1,802 results, a total number of 16 studies were included in the review.

## Surgeons Sleep Deprivation and its Effects on Dexterity

In the recent years, great effort has been devoted into developing objective measurements of technical ability in surgeons. Surgeons expertise involves a broad spectrum of skills, ranging from technical abilities to leadership. Dexterity, defined as “*the skill of performing tasks, especially with the hands*” (31), is considered a necessary requirement for an accomplished surgeon. However, there is not a unique way to objectively assess dexterity. Commonly adopted approaches are: direct-indirect observation (e.g., using video recordings) with subsequent expert evaluation of the procedure, possibly defining a priori scoring criteria (31), use of animal models and autoptic product evaluation, motion analysis of surgical instruments (32) or surgeons hands (33). Several interfering factors, both modifiable and unmodifiable, can influence dexterity: (i) low ambient and skin temperature contribute to lower dexterity (34, 35), (ii) being younger is correlated to better dexterity scores as well as being able to produce higher grip forces (36), (iii) wrong sized gloves impair dexterity (37), instead appropriate double gloves (with optimized thickness and gloves disposition) neither alter dexterity nor tactile sensitivity (38–40).

One of the first studies focused on understanding the link between surgeons dexterity and sleep was conducted by Goldman et al. (41). Specifically, the analysis of surgical performance was achieved via simultaneous video recordings and electrocardiogram (EKG) signals of the performing surgeon. Afterward, the videotapes were analyzed to evaluate surgical performance according to “*previously described criteria which included adequacy of exposure, inappropriate motions and inefficiencies due to indecisive or inflexible activity*.” In summary, 5 of the 33 surgical procedures included in the study were carried out by a surgeon who slept less than 2 h during the preceding night. The procedures conducted under SD were evaluated as “*marked with indecision*.” Moreover, the authors reported that under SD “*poorly planned maneuvers exceeded 30% of the operative time*.” This study was the first to quantify, to the best of our knowledge, a tentative link between sleep quality and surgeons' dexterity.

In surgical settings, the acquisition of many skills, either technical or not, is moving from the operating room to the surgical skills laboratory through the use of simulations, allowing the interactive performance of the trained operator in an environment that replicates a real-world clinical scenario. Simulation can include anything from the use of standardized patients and synthetic materials or animal tissues to high-fidelity advanced virtual reality (VR) systems (42). Most of the studies conducted so far on surgical dexterity under SD have employed laparoscopy simulators. However, despite a VR simulator may provide useful information about surgical performance, a perfect training model would be one closely resembling the complex reality of the operating room (43). In fact, it has been demonstrated that simulation training based on *in vivo* porcine models might lead to an ameliorations in surgical performance (44).

To this regard, the effects of SD on microvascular anastomoses, performed in a murine *in-vivo* model, were studied by Basaran et al. (45). In this study, only one experienced surgeon was recruited to conduct surgeries in 48 Wistar Hannover rats, thus representing the main limitation in this investigation. Anastomosis were performed on the proximal and distal stumps of the femoral artery, previously exposed and isolated. Many parameters were used to evaluate surgical performance, such as: (i) anastomosis time, (ii) error score calculated using the Selber checklist (46), (iii) the widely used global rating system or GRS (47), (iv) an autopsy score measured the day after the procedure, and (v) patency also tested the day after the procedure using the milking test (also known as double occlusion test). Results of the study showed that a progressive shortening of anastomosis time took place across the three sessions. The authors suggest that this could be due to an increased urge of the surgeon to conclude the procedure, anyway it cannot be ruled out that a progressive learning could have reduced the time needed to perform the experimental task. Performance measured with the Selber error checklist (46) was found to be significantly worsened by SD. More in detail, none of the scores about dexterity significantly changed, as opposed to the visuospatial ability found significantly worsened as well as the anastomotic leaking score. The overall score, assigned during the post-mortem examination, was also found significantly worsened when comparing the post-work or the SD session to the pre-call one. Overall the surgical procedure, as measured by the GRS standard (47), was found to be significantly worsened in SD condition. Moreover, the authors reported that all GRS items changed significantly in a pejorative way. The GRS score is articulated in different sub-items evaluating specific aspects of the surgical procedure. In this study the GRS sub-items evaluating the presence of repeated and unnecessary movements of the surgeon and the one evaluating the degree of time efficiency changed in a very significant (*p* < 0.001) manner.

The use of the *in-vivo* animal models has certainly several benefits, in terms of condition as it mimics conditions occurring during live surgery such as bleeding, pulsation and liquid filling of the vascular tree. Nevertheless, this method is rarely applicable because of its costs, ethical issues, and complexity of design. These reasons led to the development of various type of virtual simulators, as an objective mean of evaluation able to reproduce with a high degree of accuracy and content validity several surgical procedures or propaedeutic exercises of basic surgical dexterity (e.g., suturing). Simulation has also several drawbacks as usually it is not really linked to the daily tasks that a surgeon has to deal with (e.g., patient supervision outside the surgical theater).

A laparoscopic virtual simulator study conducted under SD pressure was designed by Taffinder et al. They directly investigated the effects of SD on surgical dexterity, taking advantage of the Imperial College Surgical Assessment Device (ICSAD), and a Mist-VR laparoscopic virtual reality simulator (48). Six residents took part in the study. Time to completion and number of errors were found to be inversely and linearly related to sleep duration. Surgeons exposed to SD made more errors (20%) compared to the undisturbed sleep condition group and spent longer procedure times (14%) compared to the undisturbed sleep condition group. Those increased impairments in performance were correlated with increases in stress and decreases in arousal levels. The recorded worsening in performance remained significant after arousal was taken into account as a covariate, further highlighting that SD induces stress as an independent variable.

Likewise, other studies have detected significant increase in the number of errors (45, 49–53). Grantcharov et al. assessed the effect on simulated surgical procedure on 14 surgery interns belonging to a gastroenterological surgical unit (50). A MIST-VR (Mentice Medical Simulation, Gothenburg, Sweden) laparoscopic surgery simulator was employed (54). The employed simulator embeds six different tasks that were selected after the study of the ergonomy of laparoscopic procedures (55). The median sleep duration during the night on call was of 1.5 h, with a range of 0–3 h. In SD conditions subjects took significantly more time to complete all task but the number 2 (all values are in seconds, 5.4 vs. 7.6, 5.6 vs. 7.8, 6.7 vs. 8.1, 15.0 vs. 18.1, and 18.2 vs. 23.8). The number of errors was significantly higher for tasks 1 and 6 (median number of errors, 0.6 v 1.0 and 1.4 v 3.5). A significant increase of the number of unnecessary movements was detected in task 5 and (7.8 v 9.4 and 6.1 v 8.2), the latter was the most complex and the suitable to better investigate the surgical performance outcome, as was previously tested in a porcine animal model (56). Another study by Eastridge et al. (51) used the same simulator and tasks adopted by Grantcharov. Thirty-five surgery residents participated. Experimental sessions were administered in a “rested pre-call” and “SD post-call” conditions. The cumulative number of errors recorded obtained by summing errors made in each of the three tasks was significantly higher in the SD condition. Economy of motion of either hands and time to complete the given tasks did not change significantly.

Another simulation study under SD was performed by Tsafrir et al. (52) who put care at monitoring and controlling possible confounding factors. A Lap Mentor (Simbionix) simulator was used. The study population included 26 residents who were divided in expert and naïve groups (those who attended at least 20 laparoscopic procedures were deemed as experts). No statistical difference between the two groups was present in terms of self-reported sleep duration, coffee consumption, handedness, gender, experience in computer or video games. After an initial supervised training phase, no significant differences in simulator's performance metrics were found between expert and naïve subjects. In both novice and expert groups, the simulation performance significantly worsened in the SD condition, specifically in efficiency (e.g., time to complete task), and safety (number of errors) performance metrics. Novices performance deterioration was significantly worse in some tasks when compared to experts. As an example, in the naïve subjects the total time to complete the task in the camera manipulation at a 30-degree was increased by 12.5% after SD (average 136 s after SD vs. 119 s during baseline), while in the experts there was a significant increase of 8.0% after SD (average 112 s after SD vs. 103 s during baseline).

All the studies reported so far have investigated the effects of acute sleep restriction-deprivation on surgical performance. However, a more realistic scenario is one in which surgeons are exposed to the effects of chronic SD rather than a single night without sleep or with restricted sleep (<1 h). In normal subjects, the cumulative effects of even a mild chronic sleep restriction (6 h of time in bed per night) can lead to neurocognitive impairments comparable to those following two consecutive nights of total SD in less than 2 weeks (8). Moreover, it has been shown that the subjective capacity to evaluate the effects of SD is limited and suffers from ceiling effects in the perception, leading the subject to underestimate the extent of the detrimental effects of sleep curtailment (8, 57). In this context, a study has been performed in order to investigate the effects of being on call for seven consecutive days (49). The study enrolled 21 surgical interns from different specialties. A MIST-VR (Mentice Medical Simulation, Gothenburg, Sweden) laparoscopic surgery simulator was employed. Before the beginning of the study, participants were trained on a series of six different exercises of growing complexity. The maximal deterioration of performance in the two surgical dexterity assessment tasks was registered after the first night on call. The time to complete each of the two tasks (time in seconds for task one: bsl 86 ± 4.5 vs. SD 100 ± 12.3 *p* = 0.025; and task two: 36 ± 9.1 vs. SD 40 ± 10.2) as well as the number of errors in the first one (task one: bsl 86 ± 4.5 vs. SD 100 ± 12.3 *p* = 0.025) was significantly increased. Interestingly, the sub-group of emergency medicine interns exhibited a constant and significant decrease in performance throughout the week as opposed to all other sub-groups. Emergency medicine interns were the ones to sleep the least (they underwent total SD during each of the seven nights, self-reported by questionnaire), to cover the highest median number of steps per day (6453, range 5042–7905) and attended the largest sample of patients (median 13, range 12–15). Other studies compared the effects of SD on simulated surgical performance with mild levels of alcohol intoxication, as the effects of this second condition on attention are known to be similar to that of SD (9). In the study of Mohtashami et al., sleep deprivation was defined as a total sleep time in the previous 24 h of <3 h. Alcohol administration was regulated to reach the legal intoxication level of >0.08% mg/mL Blood Alcohol Concentration. Nine experienced gynecologists were included in the study. Technical dexterity was measured using a physical box trainer, three laparoscopic exercises (cup drop, rope passing, pegboard exchange) of increasing difficulty were performed. The performance resulted impaired after a night with <3 h of sleep (53). The comparison of performance achieved when intoxicated with alcohol or in SD condition was found to be similar. When intoxicated subjects were faster compared to the SD condition, but their performance was significantly poorer in the most difficult task. The same difficult task was the only one in which surgical performance was considered negatively altered after a review of the task, carried on in a blind condition by 3 expert reviewers. Another study (58) compared mild SD (average sleep duration 3.75 h, range, 3–5 h) without alcohol to 0.43 mg/L BAC condition. Five surgeons were recruited and performed three different experimental sessions, baseline (no SD and no alcohol consumption), SD (only sleep restriction) and lastly a combination of SD and alcohol consumption. Each subject was evaluated three time for each of the three conditions. Surgical dexterity was evaluated using a MIST-VR laparoscopic surgery simulator. Authors reported a significant worsening of performance for all metrics (average task duration, average number of errors, average diathermy time and average diathermy time erroneously applied outside the target).

Overall this first group of studies found some alteration of surgical dexterity, assessed during simulated procedures, after SD. In parallel, a large body of experiments found no differences in simulated surgical performance after SD (59–66).

Lehman et al. carried out a study (61) to better understand the psychomotor and cognitive effects of a 24 h shift on surgical residents. The study population was divided in two groups: 17 surgical residents (test group) and 13 medical students (reference group). The reference group was included in the study to include a clear picture of possible effects of residual learning that may occur during the testing. The reference group included only medical students with no prior experience in laparoscopy. The study used a surgical simulator called Virtual Endoscopic Surgery Trainer (VEST), the apparatus embeds haptic feedback and was validated in a previous study (67). Three different tasks were selected, for each task authors calculated two performance parameters named scoreTP and scoreE. ScoreTP represented a measure of time and instrument path length, while scoreE summarized error related scores. Two standard neuropsychological tests were also included in the testing sessions, (1) an attention and concentration performance test, the d2T test, (2) and a visuoperceptual and visual attention test, the trial making test (TMT). All subjects underwent 5 training sessions of 1 h each before the beginning of the test phase to control for possible confounding factors introduced with learning. No significant performance impairment was found in the comparison of scoreTP and scoreE obtained during the pre-call session and the post-call (after SD exposure). Mean self-reported sleep duration recorded during the on-call time preceding the post-call testing was 2.9 ± 1.4 h.

Jakubowicz investigated the effects of 24 h on call on a simulated sinus surgery (66). Unlike the previous papers reviewed, the simulator chosen for this study (ES3 v2.0.2, Lockheed Martin) was developed to simulate a complete surgical procedure and not only simple psychomotor tasks. All participants were trained with the simulator before attending the actual experimental session until saturation of the learning curve. Each subject was tested twice before a 24 h on-call period and after being post-call. Overall surgical dexterity score did not decrease significantly after 24 h on call, moreover the performance metrics showed learning during the administered experimental sessions.

Veddeng et al. focused their attention on gynecologist interns (64). Twenty-eight gynecologists were enrolled in the study; three expert surgeons were used as reference for the laparoscopic task. Each of the subjects received a training session on the VR simulator. Moreover, the subjects have been divided in different sub-groups according to their level of expertise. The surgical procedure simulated was a salpingectomy. Participants skills were evaluated according to three metrics: time taken to complete procedure, the total length of instrument movements path (centimeters), and an estimation of blood loss (milliliters). A cognitive test, the Cambridge neuropsychological test automated battery (CANTAB), has been also administered before or after the VR test. The study showed no significant differences in laparoscopic skills assessed after the on-call shift compared to the baseline measurements. The CANTAB testing scores assessed after being on-call recorded significantly higher reaction times, a significant improvement in the Paired Associates Learning test.

A similar study design was adopted by Elizabeth et al. (60), who investigated the effect of SD while using an ophthalmic surgical simulator (Eyesi, VRmagic, Mannheim). Nine residents were included in the study. Each of them was tested in three different conditions: (i) pre-call (with at least 7 h of sleep during the previous 24 h), (ii) post-work (same SD condition as previous condition but after an 8 h work day), and (iii) post-call (<3 h of sleep during the previous day). No caffeine consumption was allowed during a period of 12 h preceding the test. An attempt to control for chronic SD was made, as subjects were asked to sleep at least 6 h a day during the preceding week, but no objective assessment was clearly implemented. Subject were asked to self-report wake-up time, number of hours slept during the previous night and the number of hours slept during the previous week. The average self-reported sleep duration during the three conditions of the study was: 7.6 ± 0.6, 7.2 ± 0.4, and 1.9 ± 1.2 h. No statistically significant differences were found in both simulated tasks in any condition of testing.

Another study failing to identify difference in simulated performance after SD has been performed by Yi et al., who investigated surgical dexterity and psychomotor performance before and after one night-float shift (65). Nine surgical residents undergoing a night-float rotation were enrolled in the study and assigned to one of two experimental groups (night-float and 24 h call). For the study a LAP mentor (Simbionix) laparoscopic surgery simulator was used. Subjects had to fill out a questionnaire about sleep quality and quantity in the previous week and previous night, hours slept on shift, cups of coffee or other stimulants consumed while on shift (e.g., cigarettes), number of calls from staff during the shift, as well as the Epworth sleepiness scale. Even though residents being on-call for 24 h saw more patients (28 vs. 11 for the night shift only, *p* = 0.008) and walked farther (10,731 vs. 4990 *p* = 0.0037, measured suing a pedometer) their level of fatigue and sleepiness at the end of the shift was comparable to those working a 12 h night-float shift (ESS scores 15 vs. 11, *p* = 0.14). Surgical performance was assessed using a set of metrics available from the simulator, capable of measuring the number of instruments movements, the overall accuracy, the economy of movement, the time to complete the task and the speed of movements. None of the performance metrics revealed any statistical difference between respective baseline, recorded before the beginning of the shift, and the session administered after 12 or 24 h call. It might be relevant that the baseline number of hours of sleep for both groups was as low as 5.6 h for the 12 h shift and 5.4 h for the 24 h one. Since this sleep duration is rather low, experimental results may have been confounded by the effect of the superimposed chronic sleep restriction accumulated before the study begin.

A study conducted by Olasky et al. (62) tried to understand whether experienced surgeon's performance would have been differently affected by SD, compared to less experienced colleagues. Twenty-two surgical residents and novices were asked to use two different simulators, a box trainer complaint with the Fundamentals of Laparoscopic Skills (FLS) guidelines and Virtual Basic Laparoscopic Surgical Trainer (VBLaST). Both simulators were used to study the performance in the peg transfer task, a standard pick and place exercise. To understand the level of surgical expertise, subjects were classified based on post-graduate year and self-reported relevant experience. Moreover, they were asked to fill a form about overall fatigue level (well-rested/tired) and sleep-hour during the night before the task. Results reported that there was no significant difference in sleep hours or fatigue level across experience levels (*F* = 0.98, *p* = 0.05; *F* = 0.91, *p* = 0.05). While, no correlation was found between experience level and sleep hours (*r* = 0.16, *P* = 0.05), between experience and fatigue level (*r* = −0.04, *p* = 0.05), and remarkably between sleep hours and fatigue (*r* = −0.10, *p* = 0.05). Experience was instead found to be related with a positive relationship to performance on both FLS and VBLaST simulators (*F* = 6.14, *p* = 0.022, and *F* = 6.87, *p* = 0.016).

Schlosser et al. conducted a study on 38 surgeons assessing the effect of acute partial SD on surgical performance and analysis of bio-physiological responses to the different experimental conditions (63) (prior to a 24 h call, post-call, and after 24 h of rest); this is the first study that used objective measure of fatigue. This study employed a LapSim virtual reality laparoscopic surgical simulator. All measurements (Stanford- Sleepiness Scale (SSS), Saliva Cortisol-ELISA, pupillography, and the d2 Paper-Pencil Test) were assessed at the same time of day, between 09:00 a.m. and 10:00 a.m. To avoid for possible bias introduced by different experience levels, subjects were divided in groups of homogeneous experience level. Reported sleep duration relative to the three experimental conditions were the pre-call 6.7 ± 0.16 h, post-call 4.09 ± 0.28 h, and after rest 6.47 ± 0.15 h. Experienced surgeons felt significantly less sleepy when compared to junior residents and interns in the post-call condition (SSS sleepiness scores: senior residents 2.9 ± 1.2 vs. interns 3.95 ± 0.97 and junior residents 3.89 ± 0.78), despite a similar amount of sleep post-call. No significant changes of pupillary unrest indices and saliva cortisol concentration measured pre-call were found. Although, in the post-call condition, interns' cortisol saliva concentration was significantly higher if compared with the other two groups levels (interns, 7.52 ± 4.24 ng/ml, junior residents 4.34 ± 2.6 ng/ml and senior residents 4.55 ± 4.34 ng/ml). The results of the d2 Paper-Pencil alertness test improved in a constant fashion from the first pre-call condition to the last after-rest testing session indicating a possible effect of learning occurring during the test itself. Surgical performance metrics changed significantly only for the parameters measuring economy of motion, performance scores were found to be improved in the post-call session.

Uchal et al. tried to measure the impact of SD after a 24 h on-call shift (post-call group), comparing it to a standard 8-h work shift (post-work group) (59). Product quality and procedure effectiveness were evaluated in suturing a perforated ulcer on a laparoscopic foam hollow stomach. Subjects were randomly allocated to each group. No significant differences were detected in all the parameters taken into account and the surgeons operating in post-call group did not show any worsening in performance. Neither the accuracy of movements nor the number of errors made the experimenters concluding that the SD would have interfered somehow with their tasks. It is noteworthy that this is the only randomized controlled trial (RCT) conducted. RCTs are regarded as the highest level of methodological quality. Even though this care in experimental design, this study did not find significant differences analyzing the simulated surgical procedures. Beside this control and SD groups had significantly different exposures to SD (self-reported time slept in the 24 h before the experiment, 1.5 vs. 6.5 h), hence strengthening the theoretical ability of the study to identify any difference.

Tomasko et al. compared the surgical dexterity of two groups of medical students randomly assigned to a control-rested (at least 6 h of sleep in the preceding night) condition or to SD (<2 h of sleep) (68). Thirty-one subjects were recruited for the study. Two virtual reality laparoscopic surgical simulators were employed, i.e., the RapidFire (Verefi Technologies, Elizabethtown, PA) and the EndoTower (Verefi Technologies, Elizabethtown, PA). Both simulators provided performance scores including number of errors, time to complete the task, efficiency of movement and time spent with off-axis camera. All subject underwent a preliminary phase of training. In addition to the surgical dexterity measures subjects were asked to fill the Epworth sleepiness scale and the NASA Task Load Index. Subjective sleepiness was significantly higher only in the SD group: ESS score at baseline 5.07 ± 2.79 vs. SD 12.27 ± 6.77. The between group comparison of the RapidFire simulator scores relative to the rested vs. SD groups revealed no statistically significant difference. The comparison of performance scores of the SD group recorded during rested and SD condition revealed a significant improvement of the scores during the SD test session only for the simplest task (RapidFire Level1 scores, 94.0 ± 8.4 vs. 99.7 ± 9.2, *p* = 0.003). Instead the control group registered a significant improvement only in the most difficult task (RapidFire Level3 scores, 67.2 ± 8.7 vs. 70.4 ± 8.6, *p* = 0.03). The SD group showed significantly higher NASA TLX scores in the items: total workload, frustration, performance, physical demand, and temporal demand when compared to the rested-control group. Overall SD did not alter surgical dexterity but increased the subjective mental and physical effort of the subjects to achieve comparable surgical performance scores.

## Objective and Longitudinal Tracking of Sleep and Drowsiness

Drowsiness can be measured and tracked using subjective and objective approaches, depending on the aim and contingencies dictated by the boundary conditions of experimental design, subject compliance, and so forth. A subjective assessment takes into account a personal estimation coded in a measure exploiting validated scales. The most used scale of drowsiness measurement is the Karolinska Sleepiness Scale (KSS), which consider a nine-point rating scale with verbal anchors attached to each discreet level. This kind of approach has a modest time resolution (when compared to objective instrument-based techniques). Moreover, an active participation of the subject is requested for each measure and hence a perturbation in his inner state is produced (e.g., eliciting an arousal response). Regardless these limitations, these methods have an easy application and can be used to collect data from large pools of subjects (e.g., using mail questionnaires).

Objective measures of drowsiness can be obtained using behavioral or physiological measures. The real-time monitoring of vigilance state has been extensively studied in the transportation industry (e.g., aviation, railway workers) and by using of various type of driving simulators [for in depth reviews see: (69–71)]. The standard process of vigilance state scoring requires the use of system capable of recording six EEG channels, two channels electrooculogram, and two-channels electromyogram (72).

Among behavioral measures of sleepiness, simple reaction times are well known to be affected by SD and sustained wakefulness [e.g., (8)]. The standard version of the PVT test lasts exactly 10 min, although shorter variants of 3 min were studied and compared to the longer one (19). As subjects are required to actively carry on the task itself, this technique might be unappropriated where absolute unobtrusiveness is required.

Physiological measures can be used to detect, measure and track drowsiness and sleep. Miniaturized single channel EEG systems were able to distinguish with high accuracy [98.3 ± 4.1% vs. standard AASM EEG scoring (72)] sleep-drowsiness in aviation pilots during long-haul flights (10 ± 2.0 h) (73). Another algorithm reached 83.6% of accuracy in detecting drowsiness using a neural network model fed with 7 descriptors extracted from wavelet analysis of spectral data calculated from three EEG channels (74). It has also been proposed to use 1D convolutional neural networks to create predictive models of drivers cognitive performance starting from EEG data (75). It is also interesting to note that some of the available drowsiness detection systems are computationally efficient and can be used in real-time systems. This kind of approach could be effectively replicated in studying surgeons during night-shifts or while sustaining long sleep curtailment periods, in order track sleepiness. Currently, there are various proprietary and open systems that can be used and/or adapted to the surgical scenario in order to estimate vigilance state (76) and workload metrics (77, 78). Other physiological measures of drowsiness have been repeatedly shown to be useful for predicting drowsiness in specific context, such as (i) PERCLOSE (79, 80), (ii) blinking rate, (iii) eyelid movements (81), (iv) head nodding, (v) leap stretch (82, 83), and so forth slow eye movements were shown to correlate with EEG theta and delta power band as well as with the nadir tympanic temperature and subjective sleepiness (84). Although these relations were proven to be robust only in a closed eye condition, hence limiting severely the usefulness of this metric in the field. Some of these physiological markers of drowsiness can be used to effectively prevent accidents caused by low vigilance (85). Also urinary melatonin and cortisol in saliva were found to be significantly different when comparing pre-call with post-call values (86).Hearth rate variability is another physiological biomarker that can be used to track SD effects on alertness and reaction times (87). The power spectrum of RR-interval in the frequency between 0.02 and 0.08 Hz classified subject performance at the PVT with sensitivity and specificity similar to that of PERCLOSE and superior to that recorded using EEG power spectrum metrics. RR power in the aforementioned frequency band correlated with the 40 h trend of PVT lapses.

An indirect source of information regarding sleepiness and drowsiness may come from the longitudinal monitoring of sleep schedule through the use of Actigraphy. Actigraphic recordings also shows that during the on-call day and in the first day post call sleep time was significantly increased. In the first post call day, actigraphic activity was recorded to be at the lowest level. Actigraphy can be used to track sleep non-invasively over long time span (88). This technique has been used to track and measure the exposure of the SD in surgeons and other groups particularly exposed to SD (e.g., the military, pilots, transportation industry). McCormick et al. used actigraphic data to objectively track sleep duration and timing (89). The mean sleep duration was 5.3 h with mean individual average sleep time than spanned from 2.8 to 7.2 h. The study used sleep data to feed the so-called sleep, activity, fatigue, and task effectiveness (SAFTE) model that can be used to estimate the individual performance of certain cognitive domains (e.g., attention, reaction times, decision making) (90). They found that residents were fatigued during waking state for 48% and impaired during 27%.

Moreover, the spectrum of non-medical devices validated against medical equipment counterparts, or at least developed aiming to track sleep variables, is constantly growing. As current literature is still relaying commonly on subjective evaluations of sleep metrics, such non-medical devices may add valuable information to future research. Anyway, to guarantee the reliability and reproducibility of measures, non-medical devices used for research purposes should be validated for the specific application of sleep monitoring before being enrolled in actual research studies [e.g., (91, 92)].

## Countermeasures Against Detrimental Effect of SD

As a general guideline to ensure optimal performance throughout the work shift, proper sleep hygiene, and napping scheduling should be used. There are meta-analytic evidences suggesting that napping is an effective counter measure to mitigate the adverse effects of SD (93). Short naps (5–15 min) can positively affect vigilance in the next 1–3 h (94) and are not followed by significant sleep inertia (SI) impairments (95, 96), as they almost do not show slow wave sleep. Naps longer than 30 min can produce positive effects on cognition for several hours. The effects of SI should be thoughtfully considered when scheduling naps in an operational setting (97). Dissipation of SI detrimental effects over time is thought to happen within 20 min from awakening (98) but is affected by a multitude of factors. Among them: the amount of slow wave sleep during the nap, the actual sleep stage while being awaken, circadian point. Interactions of these factors may worsen SI detrimental effect and determine a lengthening of SI effect over time. Suddenly waking up due to an emergency response might lead to act while in an altered state due to SI, perhaps affecting both cognitive and motor sphere (97). In a population of surgery residents, the administration of a self-regulated length nap, had significant positive effects on executive skills (99). The nap group slept on average 147 ± 71.0 min during the intervention night and recorded a better performance in a task switching test and better reaction times measured using the standardized GoNoGo task. While programming napping, another parameter that can be optimized is nap timing. To attain the best positive effects, already SD individuals should take naps as sleep pressure begins to rise, while rested individuals take more benefits in extending waking as long as sleep pressure is not an issue (100). Concerning sleep hygiene, it has been shown in a population of interns, that there is an inverse correlation between total sleep duration and the amount of work hours (11). Moreover, it was demonstrated that optimizing the work schedule by removing night shift of more than 16 h and reducing the total amount of work to 80 h per week, resulted in a reduction of the number of attentional lapses during work and of the number of serious medical errors in an intensive care unit (4).

In the context of sleep and performance optimization, the circadian modulation of sleep propensity should also be considered. This due to the evidence that some part of the day are associated to physiologically different sleepiness and sleep propensity (101), knowing this work schedule can be done in order to avoid peaks of sleepiness and also try to exploit naturally low sleepiness periods to address complex and difficult tasks. In context were scheduling is unpractical or impossible to implement, bright light therapy could be used to counteract at some extent the endogenous drive to sleep (102). Another approach might be the optimization of sleep quality during naps or nocturnal sleep. As an example, subject exposed to pink noise while sleeping (103) demonstrated better sleep quality measured as a significantly lower sleep fragmentation.

In specific situations of emergency or when fatigue avoidance and a sleep optimization approach cannot be adopted, a possible alternative countermeasure that could be adopted to temporarily alleviate the effects of SD on cognition is the use of stimulants and wake promoting drugs.

A widely available substance acting as a wakefulness promoter is caffeine [for an in depth review on the topic e.g., (104)]. Caffeine intake was shown to reduce sleepiness and to reduce waking EEG theta activity and 0.75–2 Hz band during recovery sleep after SD even if caffeine saliva concentrations were extremely low (below the detection limit for some subjects, population average concentration 1.8 ± 1.0 μmol/l) (105). The relationship between caffeine dosage and response is still unclear, attention performance seems to be affected even with relatively low doses but it might reach maximal effect with middle-high doses of about 200 mg (106). Associated to this very same middle-high dosage, caffeine can induce several undesired side effects that can potentially harm surgical procedures, such as: anxiety, nausea (107) and more importantly for dexterity, an increase of hands tremors: in surgeons 200 mg of caffeine were found to enhance tremor by 33% (108, 109). While it is recognized that caffeine enhances performance in simple tasks, its impact in complex tasks is still controversial. Moreover, the caffeine effects need to be considered in pre-dose level of arousal (110, 111). Caffeine effect is also known to be mediated by specific genetic variations. A variation of the adenosine deaminase (112), expected to be occur in 8–12% of individuals, was found to affect both sleep and waking EEG. Subject with the mentioned variation showed higher amplitude delta oscillations as well as theta-low alpha activity. The latter two EEG frequency bands are known biomarkers of sleepiness and overall homeostatic sleep regulation (113, 114). Moreover, it was shown that subjective sensitivity to caffeine effects are paired with significantly different responses to SD (115). Adenosinergic mechanisms were found to modulate changes in both behavioral measures of the effects of sleep deprivation (specifically using the PVT) and on the same EEG frequency bands mentioned before. Moreover, subjects showing more marked detrimental effects of SD where also those to obtain the greater benefits (as shown by PVT) from caffeine consumption, independently from their sensitivity to caffeine.

A study conducted by Aggarwal et al. focused on the understanding of how the administration of caffeine plus taurine may affect the surgeon's performance on the Minimally Invasive Surgical Trainer VR (MIST-VR), in sleep deprived condition (116). Authors compared rested condition (baseline), with SD and placebo administration and SD with the supplementation of caffeine and taurine as stimulants support. Eighteen medical students were recruited. Participants underwent a preliminary phase of supervised training using the laparoscopic simulator. Subjects were randomly allocated in one of the three study arms: (i) SD-placebo (vitamin C and calcium tablets), (ii) SD-caffeine (150 mg), and (iii) SD plus caffeine and taurine (150 mg + 2 g, respectively). During the experimental sessions, the various experimental tasks were administered in the following order (i) surgical simulator assessment, (ii) PVT, stroop task, (iii) Wisconsin card sorting test, and (iv) mental arithmetic test. Surgical simulator performance scores significantly worsened in SD-placebo condition when compared to baseline (time taken to complete the task, median 41 vs. 35 s; *p* = 0.016; economy of movement 3.25 vs. 2.95 m; *p* = 0.016; number of errors 66 vs. 59; *p* = 0.021). When only caffeine was given in the experimental manipulation, there was no statistical difference between simulator metrics pertaining to the baseline and SD plus caffeine conditions, except for the number of errors of the manipulate diathermy task that were significantly higher in the SD-placebo condition (63 vs. 59; *p* = 0.046). PVT recorded reaction times were significantly longer in SD-placebo condition (377 vs. 299 ms; *p* = 0.008), in the caffeine and caffeine plus taurine conditions reaction times were not different from the one recorded in the baseline condition (307 vs. 299 ms; *p* = 0.214 and 326 vs. 299 ms; *p* = 0.110, respectively). Subjective sleepiness was higher for all conditions including those with experimental manipulation. Scores obtained in the Stroop, WCST and mental arithmetic task did not change significantly in any of the experimental conditions. The data collected in this study suggest that the lack of sleep negatively influences simulated laparoscopic psychomotor skills. This hold true even though the administration of psychostimulants, such as caffeine and taurine.

Among stimulants drugs, modafinil is used for the treatment of excessive sleepiness syndrome in shift work (117) and in narcolepsy (118). Particularly important in surgeons performance, this drug does not induce tremors, anxiety and nausea, that are instead associated to high dosages of caffeine (107). Moreover, it has been observed that the administration of 200 mg of modafinil in SD physicians can improve the performance at a CANTAB neurophysiological test but not in basic procedural tasks (119).

## Conclusion and Discussion

Current evidence emerging from the literature does not provide a compelling ground to draw consistent conclusions about the effects of SD on surgical dexterity. A number of studies found no significant variations following either acute SD or sleep restriction (59, 61, 62, 64–66, 68, 116, 120), while others found a worsening of quantity and quality performance (45, 48–52) or even improvement in SD condition (63, 121). More specifically, a technical limitation in a systematic overview concerns the array of heterogeneous metrics of performance used to evaluate dexterity, hence preventing a robust statistical analysis or meta-analysis. Moreover, the inclusion criteria concerning the extent of sleep deprivation vary considerably between studies and some of these criteria overlap with the broader condition of fatigue, resulting not only from the lack of sleep. Unfortunately, no study in the field included sleep extension as a control condition, further limiting the available experimental tools for the investigation of sleep-related performance. Sleep extension could be implemented asking experimental subjects to comply to a predefined daily time in bed duration of about 10 h for several consecutive days. The well-established PVT could be paired with this sleep extension approach to objectively measure and define sleep extension duration for the specific experimental population. Such experimental methodology can dissipate previous detrimental effects of chronic sleep restriction and acute SD on neurobehavioral and physical performances (122). A good example of the use of this methodological approach can be found in (123) were sleep extension was paired with the study of objective and subjective sleep metrics and to the assessment of both cognitive and motor spheres. Observed inconsistency in results could be also explained by several other factors (see Table 1 for more details). A methodological issue found in many studies is the lack of objective and longitudinal assessment of sleep quantity and quality, either before the study onset and across its execution. Sleep metrics are often measured relying only on subjective methods (e.g., questionnaires), which are known to be relatively unreliable estimators of the effects of SD (8, 57, 124). Moreover, the extent of chronic sleep restriction, which sums up to the usually acute SD imposed in many experimental designs, is usually not measured or taken into account. This can lead to the underestimation of the contribution of over imposing chronic sleep restriction, thus confounding any sleep-related measure on performance. Moreover, a possible ceiling effect in performance could limit changes in surgical dexterity-performance as the chronic exposure to SD could impair the baseline performance, thus masking the acute SD effects. Actual exposure of experimental subjects to SD (e.g., calculating the percentage of waking during the SD phase using an objective examination such as polysomnography) and the effect of brief episodes of sleep during the SD phase, are key variables and should be collected and properly analyzed in future work. To solve this issue, we discussed several available methods to track SD and drowsiness (see section Objective and longitudinal tracking of sleep and drowsiness). Specifically, literature about fatigue modeling on the workplace gives many valuable and detailed clues on how to properly track and measure SD related risk in safety critical workplaces (125, 126). The need for non-invasive, longitudinal and objective tracking of SD should also be considered as a standard feature for future studies, with the specific aim of gathering normative data about sleep hygiene and SD in surgeons, which are now lacking. Good examples of the use of objective measures to track sleep among physicians are the studies of Basner et al. (20), and the one of McCormick et al. (89). The aforementioned methods could be implemented in future experimental paradigms where surgical errors, defined a priori as violations of approved procedural checklists, should also be included. In line with an approach already successfully implemented in the investigation after aviation accidents (127). This methodological care could pave the way to a better understanding of the relationships between one surgeon's sleep history and medical errors. Remarkably only one study (49) directly assessed the effects of chronic sleep restriction on surgical dexterity. Since sleep restriction is the most prevalent condition to which subjects are exposed, future research should focus specifically on implementing experimental paradigms useful to shed light on the effects of this condition on surgical dexterity.

**Table 1:** An overview of studies included in the review reporting the main conclusions and an analysis of the confounding factors controlled for.

**References**	**Sample size and findings**	**Surgical dexterity task or VR simulator**	**Experimental sessions**	**Statistical power for null show**	**Circadian point**	**Learning**	**Chronic sleep debt**	**Objective sleep measure**	**Stimulants intake**	**Actual vs. historical controls**	**Randomization**
Basaran et al. (45)*	***↓***1 surgeon who executed 48 procedures. Overall the surgical procedure, as measured by the GRS standard, was found to be significantly worsened in SD condition.	Microvascular anastomoses performed in a murine animal model.	Baseline testing at 08:00 a.m., vs. 01:00 a.m. of the following night, vs. 09:00 a.m. after one night of total sleep deprivation.	Yes	No	Yes	No	No	No	Actual	NA
Eastridge et al. (51)	**↓**35 surgical residents. The cumulative number of errors recorded was significantly higher in the SD condition. Economy of motion of either hands and time to complete the given tasks did not change significantly.	Virtual laparoscopy simulator: MIST-VR.	Baseline and SD testing at 8:00 and 11:00 a.m.	Yes	No	Yes	No	No	No	Actual	No
Elizabeth et al. (60)	**↔** 9 ophthalmology residents. In acute SD condition no statistically significant differences were found in simulated tasks.	Virtual laparoscopy simulator: Eyesi.	Baseline testing at 9:00–11:30 a.m. vs. 5:00–8:00 p.m vs. 9:00 a.m.−6:00 p.m. following day.	No	No	Yes	Yes	No	Yes	Actual	No
Grantcharov et al. (50)	**↓** 14 surgical residents. Surgeons took more time to complete five of the six tasks. They also made more errors and did a higher number of unnecessary movements.	Virtual laparoscopy simulator: MIST-VR.	Baseline testing during normal daytime work hours vs. testing at 09:30 a.m. after a night on-call.	Yes	No	Yes	No	No	No	Actual	No
Jakubowicz et al. (66)	**↔** 8 surgical residents. Overall surgical dexterity score did not decrease significantly after 24 h on call, moreover the performance metrics showed learning during the administered experimental sessions.	Virtual laparoscopy simulator: ES3 v2.0.2, Lockheed Martin.	Baseline testing before beginning a 24-h on call period and after its conclusion, no predefined time points for testing were reported.	No	No	Yes	No	No	Yes	Actual	No
Leff et al. (49)	↓ 21 surgical and allied disciplines residents. A significant worsening of performance (higher time to complete task and higher number of errors) was recorded during the first shift of sequential night shifts.	Virtual laparoscopy simulator: MIST-VR.	Experimental sessions administered during seven successive night shifts compared to rested baseline and with follow-up assessment.	Yes	No	Yes	No	No	Yes	Actual	No
Lehmann et al. (61)	**↔** 17 surgical residents and 13 medical students. No significant surgical dexterity impairment was found in the comparison of surgical simulator scores.	Virtual laparoscopy simulator: Virtual Endoscopic Surgery Trainer (VEST).	Baseline testing administered during four experimental sessions at 08:00 and 16:00 of on-call day vs. sleep deprivation 08:00 post-call vs. 08:00 rested follow-up.	Yes	No	Yes	No	No	No	Actual	No
Mohtashami et al. (53)*	**NA** 9 subjects. The comparison of performance achieved when intoxicated with alcohol or in SD condition was found to be similar.	Physical simulation with box trainer.	A session in sleep deprived condition (<3 h day before) was compared to another while legally intoxicated with alcohol (>0.08 mg/mL blood alcohol concentration). All sessions administered between 08:00 and 11:00.	No	No	No	No	No	No	Actual	No
Olasky et al. (62)*	↔ 22 surgical residents and attendings. Surgical dexterity measured using a peg transfer task was not affected by SD.	Physical simulation with box trainer Fundamentals of Laparoscopic Skills (FLS) and the Virtual Basic Laparoscopic Surgical Trainer (VBLaST).	Single experimental session with no rested baseline testing.	NA	No	No	No	No	No	NA	No
Schlosser et al. (63)	**↑** 38 surgical residents. Surgical performance metrics changed significantly only for the parameters measuring economy of motion, performance scores were found to be improved in the post-call session. No significant changes of pupillary unrest indices and saliva cortisol were found after SD.	Virtual laparoscopy simulator: LapSim.	Baseline testing vs. after 24 h call vs. follow-up after 24 h of rest all sessions administered between 09:00 and 10:00.	Yes	No	No	No	No	No	Actual	No
Taffinder et al. (48)	**↓** 6 subjects. Surgeons exposed to SD made more errors compared to the undisturbed sleep condition group and spent longer procedure times compared to the undisturbed sleep condition group.	Virtual laparoscopy simulator: MIST-VR.	Baseline testing between (17:00 and 18:00) vs. three conditions (08:00 to 09:00) (1) undisturbed sleep (2) sham night on-call (3) total sleep deprivation.	No	No	Yes	No	No	No	Actual	No
Tomasko et al. (68)	**↔** 31 medical students. No alterations of surgical dexterity but increased subjective mental and physical effort to achieve comparable scores.	Virtual laparoscopy simulators: RapidFire (Verefi Technologies, Elizabethtown, PA) and the EndoTower (Verefi Technologies, Elizabethtown, PA).	Baseline testing vs. after a 24 h shift in two randomized conditions: (1) sleep deprivation (<2 h of sleep) 2) rested re-test (≥6 h of sleep).	No	No	Yes	No	No	No	Actual	Yes
Tsafrir et al. (52)*	**↓** 26 surgical residents. Surgical dexterity measured with simulation performance significantly worsened in the SD condition (regarding both time to complete task and number of errors.	Virtual laparoscopy simulator: LAP Mentor Simulator (Simbionix, Cleveland, OH).	Baseline testing vs. sleep deprivation. Order was randomized.	Yes	No	Yes	No	No	Yes	Actual	No
Uchal et al. (59)	**↔** 32 post-call surgeons and 32 post-work. No significant differences were detected in all the surgical performance parameters taken into account.	Virtual laparoscopy simulator: MIST-VR.	Baseline vs. after 8 h work day or vs. sleep deprivation after 24 h on-call.	Yes	No	No	Yes	No	No	Actual	Yes
Veddeng et al. (64)*	**↔** 28 gynecologists. The study showed no significant differences in laparoscopic skills assessed after the on-call shift. The CANTAB testing scores assessed after being on-call recorded significantly higher reaction times, a significant improvement in the Paired Associates Learning test.	Virtual laparoscopy simulator: SimSurgery Educational Platform (SimSurgery AS, Oslo, Norway).	Baseline testing between 07:30 and 10:30 vs. sleep deprivation administered at 09:00.	No	No	Yes	No	No	Yes	Actual	No
Yi et al. (65)*	**↔** 9 general surgery residents. None of the surgical performance metrics revealed any statistical difference after 12 or 24 h call.	Virtual laparoscopy simulator: LAP Mentor Simulator (Simbionix, Cleveland, OH).	Baseline vs. sleep deprivation after a 24 h call vs. sleep deprivation after a 12 h call.	No	No	No	No	No	Yes	Actual	No

*Marked with an asterisk, papers published in the last 5 years. The effect of SD on surgical dexterity is simplified with the following symbols: no effect **↔**, detrimental effect **↓**, positive effect **↑**, NA, not applicable. Statistical power for null shown: express if a statistical power analysis was explicitly reported in the study; this to prove that the statistical analyses performed in the study had enough statistical power to have the ability to detect significant negative changes*.

Another common confounding factor frequently not taken into account is the use of stimulants such as caffeine (see Table 1), which can by itself alter neurobehavioral performance and attention, as debated in detail in section Countermeasures Against Detrimental Effect of SD. Caffeine effects on surgical dexterity is still a matter of debate, as specifically reviewed in Fargen et al. (109), and needs more research to be safely and consciously used in the field. In those cases where the use of caffeine cannot be avoided, its uptake should be considered as a confounding factor, given the power of new emerging modeling tools capable of predicting caffeine effects on cognition and attention (128, 129).

It is also well-know that resistance to SD effects is a trait-like individual characteristic (130), and that some genetic polymorphisms modulate the individual neurobehavioral response to the effects of sleep curtailment and deprivation (131–135). Understanding genetic coding of subjective SD vulnerability could be useful to identify the most vulnerable subjects, enabling early intervention to optimize work schedule and sleepiness of these individuals according to their physiological characteristics.

A relevant open research question that should be addressed in future studies is whether it exists a difference in the effects of SD between groups of experienced and naïve subjects. Some studies addressed directly this question (6, 52, 63). A significantly worse surgical performance only of the unexperienced group (*n* = 14 vs. *n* = 12 for the experienced group) was found under SD by Tsafrir et al. The study of Lehman and associate found no differences in the SD condition between experts and naïve subjects (*n* = 9 and *n* = 8, respectively). In the same study the two groups of subjects scored differently only in the first two of the five training sessions that took place before the baseline assessment. Same conclusion obtained in the study of Schlosser et al. where no differences in surgical performance score was found between inters (*n* = 19), junior (*n* = 9), and senior residents (*n* = 10) sub-groups in any of the experimental condition. In the study of Olasky et al., participants experience was classified using Post Graduate Year (PGY), there were 5 PGY1, 5 PGY2, 2 PGY3, 3 PGY4, 3 PGY5, and 3 surgical fellows. Experience correlated significantly only with surgical performance scores. No difference in sleep hours or fatigue level were found for each level of experience. The rather small sample size of the groups in these studies may have hampered the capacity to reach an adequate statistical power, although an a priori formal testing of this hypothesis is difficult as the effect size for the experience variable is largely unknown. Two other studies (66, 136) reported in their discussion the hypothesis of a higher impact of SD on naïve subjects. Perhaps this hypothesis could be supported by evidence coming from basic research that highlighted the relevance of sleep in motor learning. Sleep may play a role in mediating the changes needed to link neuron-scale behavior to the actual movement (137–139). Sleep is thought to contribute to establish favorable plastic changes that ultimately link sparse neural activation patterns to specific motor behaviors (140). Moreover, during sleep consolidation of motor engrams may occur (137, 138), determining an offline learning process that ultimately enhance learned motor skill. Thus, exposure to SD should be actively reduced during the initial phase of training of interns, as motor learning may be happen sub-optimally as well as academic performance (141).

Experience, and especially the effects of learning, may also be a confounding factor in measures of surgical dexterity. A simple solution to this problem is to enroll in future studies only proven experienced surgeons. A drawback of this approach may be the paucity of such skilled surgeons able to join such studies. Alternatively, authors should rely on validated and repeatable training curricula [e.g., laparoscopy (142), robotically assisted surgery (143)] to ensure that the saturation of the learning curve of the study task is reached by each study participant before the actual experimental trials. As different experimental tasks may show different learning curves, the careful selection and-or aggregation of experimental tasks should be carefully taken into account during experimental protocol definition. Regarding learning, several studies included in this review opted to include various form of training procedures (48–52, 60, 61, 64, 66, 68) or recruited experienced surgeons (45), while others did none of the previous (53, 59, 62, 63, 65). All studies that did not employed training of participants either failed to find any difference between the SD and baseline condition or recorded better performances after SD. It is noteworthy that even using scheduled training some studies found no difference while subjects were SD. Hence, it may be speculated that different learning curves may interact in different ways with task training and more importantly with the effects of SD. To counteract this confounding factor associated with “on-line” learning, future studies should use only reproducible surgical dexterity measures taken with validated surgical simulators [e.g., Lap Mentor (144), LapSim (144),MIST-VR (55, 56), Eyesi (145, 146)] or during *in-vivo* procedures using reproducible and standardized animal models.

Surgical population may also suffer from a self-selection bias as individuals, who are particularly vulnerable to SD, do not pursue surgical specialization or intentionally avoid participating in this kind of studies. Taken together, these lines of evidence may play a role in population selection of many studies within the perimeter of this field and significantly bias the results. Some of the subjective peculiarities in the response to SD can be tracked and measured using objective data, e.g., using the widely validated PVT task. Some bio-mathematical models of alertness (built on PVT data) can account for individual variability in performance (147, 148) and hence could be progressively used and developed in order to classify subjects as resistant or vulnerable to SD effects, within certain skill competencies. Moreover, this kind of models should be included in the development of novel or experimental work schedules, has they provide a safe way to simulate the impact of various schedules on physicians performance (120), thus emphasizing the managerial implications.

From SD literature we know that higher levels of SD (either chronic or acute) are linked to raised state instability (149). Several fMRI studies showed that SD is linked to a reduction of the signal coming from the dorsolateral prefrontal cortex (150, 151), an anatomical area particularly important for the execution of sustained attention tasks. This reduction in cortical activity was also found during visuo-spatial attention tasks in the intraparietal sulcus and extrastriate visual cortex (150). During prolonged wakefulness a progressive raising of theta and delta EEG power can be observed (152), this change happens together with a modification of cortical and corticospinal excitability (153). The increase in EEG theta band power can be observed in both waking and sleep EEG, suggesting that sleep homeostasis processes affect in a closely related fashion both sleep and wake. Moreover, the raised theta power could underlie compensating mechanisms against the effect of prolonged wakefulness. During wakefulness the raised theta power can be used as an objective measure of sleepiness (152). Cortico-spinal excitability and standardized motor threshold were measured experimentally using transcranial magnetic stimulation (153), in SD conditions a cortical excitability was found to be lowered and a raise of the standardized motor threshold was observed. In the same study authors were able to link the raised power of delta and theta EEG activity with the cortical excitability. It is also noteworthy to mention that sleep can also occur locally even if it is generally considered a global state of the brain (154). Local sleep occurs when part of the brain is transitioning to a more sleep like state, regardless the conscious and awake state of the subject. Recent findings, based on intracerebral recordings in humans, showed local and selective alteration of single neuron spiking after SD (155).

Moving further to the analysis of dexterity and fine movements, we did not find a comprehensive agreement between studies on the effects of SD on these skills. The bimanual fine motor coordination measured using the Purdue pegboard task was found to be impaired during SD in one study (156); however, it was observed substantially unchanged in another one where pegboard testing changed significantly only in the bimanual subtask (157). Another study using the O'Connor dexterity apparatus (158), found that after a 24-h call, finger dexterity changed in a pejorative way. A previous study used a comparable hand-eye coordination test but no differences were detected in the scores obtained across the two conditions (159). Talking about movement dexterity *per se*, we found that the majority of studies (5 studies out of a total of 6), that used the MIST-VR virtual laparoscopy simulator (see Table 1), found worsening in objectively measured surgical dexterity. Putting things in perspective, the overall number of studies that found worsening of surgical dexterity were 6 out of 16 included studies, for a comparison 8 out of 16 did not find any significant difference. The MIST-VR simulator is characterized by a steep learning curve, can differentiate surgeons vs. non-surgeons users (55) and provides surgically relevant metrics validated in animal models (56). It should be then considered to investigate in deep why such a simulator was able to measure significant differences in surgical dexterity while others did not. To further investigate these topics, the interested reader may consult a comprehensive reviews of currently available VR surgical simulators (160) and embedded tasks classification (161). Another major point to understand, as partially explained earlier, is the impact on final measurement of subject experience and expertise level, that may be mitigated by the relative short time needed to familiarize with the MIST-VR system (55).

Overall, the available literature does not provide a clear understanding of the effect of sleep deprivation on surgical dexterity. Available research presents inconsistencies in the measures of surgical dexterity and in the experimental designs employed. Known confounding factors acting on surgical dexterity measures, such as surgical expertise level, use of stimulants and possibly circadian performance variations are not consistently and objectively measured and included in the analysis of the results. Moreover, current studies did not directly and objectively measured sleep quantity and, also importantly, quality metrics, relying instead on subjective sleep measures that are known to be affected by subjective bias. These heterogeneities prevent the possibility to perform a meta analytical analysis of the available studies in the field, hence preventing us to achieve an objective understanding of the effects of sleep deprivation on surgical dexterity, thus encouraging further investigations.

## Author Contributions

TB and EC performed literature review and wrote the manuscript. PdA, PD, and AM participated with insightful discussions during preparation and manuscript revisions. UF and GC supervised the entire review activity and contributed in the manuscript organization of contents, actively participating with insightful discussions during manuscript preparation and revisions.

### Conflict of Interest Statement

The authors declare that the research was conducted in the absence of any commercial or financial relationships that could be construed as a potential conflict of interest.

## Abbreviations

ACGME: Accreditation council for graduate medical education
BAC: Blood alcohol concentration
CANTAB: Cambridge neuropsychological test automated battery
EEG: Electroencephalography or Electroencephalogram
EKG: Electrocardiogram
FLS: Fundamentals of laparoscopic skills
ICSAD: Imperial college surgical assessment device
GRS: Global rating system
KSS: Karolinska sleepiness scale
NREM: Non-REM
PVT: Psychomotor vigilance task
SI: Sleep Inertia
SD: Sleep deprivation
SSS: Stanford sleepiness scale
TMT: Trial making test
VEST: Virtual endoscopic surgery trainer
VR: Virtual reality
WCST: Wisconsin card sorting.

## References

[1] Krause AJ Simon EB Mander BA Greer SM Saletin JM Goldstein-Piekarski AN. The sleep-deprived human brain. Nat. Rev. Neurosci. (2017) 18:404–18. 10.1038/nrn.2017.55 28515433 PMC6143346

[2] Hinz A Glaesmer H Brähler E Löffler M Engel C Enzenbach C. Sleep quality in the general population: psychometric properties of the Pittsburgh Sleep Quality Index, derived from a German community sample of 9284 people. Sleep Med. (2017) 30:57–63. 10.1016/j.sleep.2016.03.008 28215264

[3] Barger LK Ayas NT Cade BE Cronin JW Rosner B Speizer FE. Impact of extended-duration shifts on medical errors, adverse events, and attentional failures. PLoS Med. (2006) 3:2440–8. 10.1371/journal.pmed.0030487 17194188 PMC1705824

[4] Landrigan CP Rothschild JM Cronin JW Kaushal R Burdick E Katz JT. Effect of reducing interns' work hours on serious medical errors in intensive care units. N. Engl. J. Med. (2004) 351:1838–48. 10.1056/NEJMoa041406 15509817

[5] Rosen IM Gimotty PA Shea JA Bellini LM. Evolution of sleep quantity, sleep deprivation, mood disturbances, empathy, and burnout among interns. Acad Med. (2006) 81:82–5. 10.1097/00001888-200601000-00020 16377826

[6] Nurok M Czeisler CA Lehmann Soleymani L Sleep deprivation, elective surgical procedures, and informed consent michael. Perspective. (2010) 363:1–3. 10.1056/NEJMp1007901 21190452

[7] Lockley SW Barger LK Ayas NT Rothschild JM Czeisler CA Landrigan CP. Effects of health care provider work hours and sleep deprivation on safety and performance. Jt Comm J Qual Patient Saf. (2007) 33:7–18. 10.1016/S1553-7250(07)33109-7 18173162

[8] Van Dongen HPA Maislin G Mullington JM Dinges DF. The cumulative cost of additional wakefulness: dose-response effects on neurobehavioral functions and sleep physiology from chronic sleep restriction and total sleep deprivation. Sleep. (2003) 26:117–26. 10.1093/sleep/26.2.117 12683469

[9] Williamson AM Feyer AM. Moderate sleep deprivation produces impairments in cognitive and motor performance equivalent to legally prescribed levels of alcohol intoxication. Occup Environ Med. (2000) 57:649–55. 10.1136/oem.57.10.649 10984335 PMC1739867

[10] Hirshkowitz M Whiton K Albert SM Alessi C Bruni O DonCarlos L. National sleep foundation's sleep time duration recommendations: methodology and results summary. Sleep Heal. (2015) 1:40–3. 10.1016/j.sleh.2014.12.010 29073412

[11] Lockley SW Cronin JW Evans EE Cade BE Lee CJ Landrigan CP. Effect of reducing interns' weekly work hours on sleep and attentional failures. N Engl J Med. (2004) 351:1829–37. 10.1056/NEJMoa041404 15509816

[12] Official Journal of the European Union *L* 299, 18 November 2003. Available online at: https://eur-lex.europa.eu/legal-content/EN/TXT/?uri=OJ:L:2003:299:TOC (accessed May 27, 2019).

[13] Landrigan CP Czeisler CA Barger LK Ayas NT Rothschild JM Lockley SW. Effective implementation of work-hour limits and systemic improvements. Jt Comm J Qual Patient Saf. (2007) 33:19–29. 10.1016/S1553-7250(07)33110-3 18173163

[14] Gander P Purnell H Garden A Woodward A. Work patterns and fatigue-related risk among junior doctors. Occup Environ Med. (2007) 64:733–8. 10.1136/oem.2006.030916 17387138 PMC2078416

[15] Lee S McCann D Messenger JC Working Time Around the World: Trends in Working Hours, Laws and Policies in a Global Comparative Perspective. London: Routledge (2007) p. 1–220. 10.4324/9780203945216

[16] Ayas NT Barger LK Cade BE Hashimoto DM Rosner B Cronin JW. Extended work duration and the risk of self-reported percutaneous injuries in interns. JAMA. (2006) 296:1055. 10.1001/jama.296.9.1055 16954484

[17] Barger LK Cade BE Ayas NT Cronin JW Rosner B Speizer FE. Extended work shifts and the risk of motor vehicle crashes among interns. N Engl J Med. (2005) 352:125–34. 10.1056/NEJMoa041401 15647575

[18] Drummond SPA Bischoff-grethe A Dinges DF Ayalon L Mednick SC Meloy MJ. The neural basis of the psychomotor vigilance task. Sleep (2005) 28:1059–68. 10.1093/sleep/28.9.1059 16268374

[19] Basner M Dinges DF. Maximizing sensitivity of the psychomotor vigilance test (PVT) to sleep loss. Sleep. (2011) 34:581–91. 10.1093/sleep/34.5.581 21532951 PMC3079937

[20] Basner M Dinges DF Shea JA Small DS Zhu J Norton L. Sleep and alertness in medical interns and residents: an observational study on the role of extended shifts. Sleep. (2017) 40:zsx027. 10.1093/sleep/zsx027 28329124 PMC5806581

[21] Arnedt JT Owens J Crouch M Stahl J Carskadon M. A neurobehavioral performance of residents after heavy night call vs after alcohol ingestion. JAMA. (2005) 294:1025–33. 10.1001/jama.294.9.1025 16145022

[22] Benson M Grimes I Gopal D Reichelderfer M Soni A Benson H. Influence of previous night call and sleep deprivation on screening colonoscopy quality. Am J Gastroenterol. (2014) 109:1133–7. 10.1038/ajg.2014.28 24980883

[23] Zohar D Tzischinsky O Epstein R Lavie P. The effects of sleep loss on medical residents' emotional reactions to work events: a cognitive-energy model. Sleep. (2005) 28:47–54. 10.1093/sleep/28.1.47 15700720

[24] West CP Huschka MM Novotny PJ Sloan JA Kolars JC Habermann TM. Association of perceived medical errors with resident distress and empathy: a prospective longitudinal study. JAMA. (2006) 296:1071–8. 10.1001/jama.296.9.1071 16954486

[25] Sturm LP Windsor JA Cosman PH Cregan PC Hewett PJ Maddern GJ A systematic review of surgical skills transfer after simulation-based training. Ann Surg. (2008) 248:166–79. 10.1097/SLA.0b013e318176bf24 18650625

[26] Sugden C Athanasiou T Darzi A. What are the effects of sleep deprivation and fatigue in surgical practice? Semin Thorac Cardiovasc Surg. (2012) 24:166–75. 10.1053/j.semtcvs.2012.06.005 23200071

[27] Parker RS Parker P. The impact of sleep deprivation in military surgical teams: a systematic review. J R Army Med Corps. (2016) 163:158–63. 10.1136/jramc-2016-000640 27625370

[28] Veasey S Rosen R Barzansky B Rosen I Owens J. Sleep loss and fatigue in residency training: a reappraisal. JAMA. (2002) 288:1116–24. 10.1001/jama.288.9.1116 12204082

[29] Amirian I The impact of sleep deprivation on surgeons' performance during night shifts. Dan Med J. (2014) 61:B4912. 25186549

[30] Gates M Wingert A Featherstone R Samuels C Simon C Dyson MP. Impact of fatigue and insufficient sleep on physician and patient outcomes: a systematic review. BMJ Open. (2018) 8:e021967. 10.1136/bmjopen-2018-021967 30244211 PMC6157562

[31] Moorthy K Munz Y. Objective assessment of technical skills in surgery. Br Med J. (2003) 327:1032–7. 10.1136/bmj.327.7422.1032 14593041 PMC261663

[32] Yamaguchi S Yoshida D Kenmotsu H Yasunaga T Konishi K Ieiri S. Objective assessment of laparoscopic suturing skills using a motion-tracking system. Surg Endosc Other Interv Tech. (2011) 25:771–5. 10.1007/s00464-010-1251-3 21072671

[33] Datta V Mackay S Mandalia M Darzi A. The use of electromagnetic motion tracking analysis to objectively measure open surgical skill in the laboratory-based model. J Am Coll Surg. (2001) 193:479–85. 10.1016/S1072-7515(01)01041-9 11708503

[34] Riley MW Cochran DJ. Dexterity performance and reduced ambient temperature. Hum Factors J Hum Factors Ergon Soc. (1984) 26:207–14. 10.1177/001872088402600207 6479983

[35] Chen WL Shih YC Chi CF. Hand and finger dexterity as a function of skin temperature, EMG, and ambient condition. Hum Factors. (2010) 52:426–40. 10.1177/0018720810376514 21077564

[36] Martin JA Ramsay J Hughes C Peters DM Edwards MG. Age and grip strength predict hand dexterity in adults. PLoS ONE. (2015) 10:1–18. 10.1371/journal.pone.0117598 25689161 PMC4331509

[37] Drabek T Boucek CD Buffington CW. Wearing the wrong size latex surgical gloves impairs manual dexterity. J Occup Environ Hyg. (2010) 7:152–5. 10.1080/15459620903481660 20017056

[38] Nelson JB Mital A. An ergonomic evaluation of dexterity and tactility with increase in examination/surgical glove thickness. Ergonomics. (1995) 38:723–33. 10.1080/00140139508925144 7729400

[39] Webb JM Pentlow BD. Double gloving and surgical technique. Ann R Coll Surg Engl. (1993) 75:291–2. 8379636 PMC2497939

[40] Fry DE Harris WE Kohnke EN Twomey CL. Influence of double-gloving on manual dexterity and tactile sensation of surgeons. J Am Coll Surg. (2010) 210:325–30. 10.1016/j.jamcollsurg.2009.11.001 20193896

[41] Goldman LI McDonough MT Rosemond GP. Stresses affecting surgical performance and learning. I. Correlation of heart rate, electrocardiogram, and operation simultaneously recorded on videotapes. J Surg Res. (1972) 12:83–6. 10.1016/0022-4804(72)90125-4 5062414

[42] De Montbrun SL MacRae H. Simulation in surgical education. Clin Colon Rectal Surg. (2012) 25:156–65. 10.1055/s-0032-1322553 23997671 PMC3577578

[43] Aboud E Suarez CE Al-Mefty O Yasargil MG. New alternative to animal models for surgical training. Altern Lab Anim. (2004) 32(Suppl. 1):501–7. 10.1177/026119290403201s80 23581125

[44] Jensen AR. Laboratory-based instruction for skin closure and bowel anastomosis for surgical residents. Arch Surg. (2008) 143:852. 10.1001/archsurg.143.9.852 18794422

[45] Basaran K Mercan ES Aygit AC. Effects of fatigue and sleep deprivation on microvascular anastomoses. J Craniofac Surg. (2015) 26:1342–7. 10.1097/SCS.0000000000001719 26080191

[46] Selber JC Chang EI Liu J Suami H Adelman DM Garvey P. Tracking the learning curve in microsurgical skill acquisition. Plast Reconstr Surg. (2012) 130:550e−7e. 10.1097/PRS.0b013e318262f14a 23018716 PMC3804357

[47] Grober ED Hamstra SJ Reznick RK Matsumoto ED Sidhu RS Jarvi KA. Validation of novel and objective measures of microsurgical skill: Hand-motion analysis and stereoscopic visual acuity. Microsurgery. (2003) 23:317–22. 10.1002/micr.10152 12942521

[48] Taffinder NJ McManus IC Gul Y Russell RC Darzi A Effect of sleep deprivation on surgeons' dexterity on laproscopic simulator. Lancet. (1998) 352:1191–8. 10.1016/S0140-6736(98)00034-8 9777838

[49] Leff DR Aggarwal R Rana M Nakhjavani B Purkayastha S Khullar V. Laparoscopic skills suffer on the first shift of sequential night shifts: program directors beware and residents prepare. Ann Surg. (2008) 247:530–9. 10.1097/SLA.0b013e3181661a99 18376200

[50] Grantcharov TP Bardram L Funch-Jensen P Rosenberg J. Laparoscopic performance after one night on call in a surgical department: prospective study. BMJ. (2001) 323:1222–3. 10.1136/bmj.323.7323.1222 11719413 PMC59995

[51] Eastridge BJ Hamilton EC O'Keefe GE Rege RV Valentine RJ Jones DJ. Effect of sleep deprivation on the performance of simulated laparoscopic surgical skill. Am J Surg. (2003) 186:169–74. 10.1016/S0002-9610(03)00183-1 12885613

[52] Tsafrir Z Korianski J Almog B Many A Wiesel O Levin I. Effects of fatigue on residents' performance in laparoscopy. J Am Coll Surg. (2015) 221:564–70. 10.1016/j.jamcollsurg.2015.02.024 26081177

[53] Mohtashami F Thiele A Karreman E Thiel J. Comparing technical dexterity of sleep-deprived versus intoxicated surgeons. J Soc Laparoendosc Surg. (2014) 18:e2014.00142. 10.4293/JSLS.2014.00142 25408601 PMC4232403

[54] Wilson MS Middlebrook A Sutton C Stone R McCloy RF. MIST VR: a virtual reality trainer for laparoscopic surgery assesses performance. Ann R Coll Surg Engl. (1997) 79:403–4. 9422863 PMC2502952

[55] Chaudhry A Sutton C Wood J Stone R McCloy R. Learning rate for laparoscopic surgical skills on MIST VR, a virtual reality simulator: quality of human-computer interface. Ann R Coll Surg Engl. (1999) 81:281–6. 10615201 PMC2503263

[56] Grantcharov TP Rosenberg J Pahle E Funch-Jensen P Virtual reality computer simulation: an objective method for the evaluation of laparoscopic surgical skills. Surg Endosc. (2001) 15:242–4. 10.1007/s004640090008 11344422

[57] Zhou X Ferguson SA Matthews RW Sargent C Darwent D Kennaway DJ. Mismatch between subjective alertness and objective performance under sleep restriction is greatest during the biological night. J Sleep Res. (2012) 21:40–9. 10.1111/j.1365-2869.2011.00924.x 21564364

[58] Kocher HM Warwick J Al-Ghnaniem R Patel AG Surgical dexterity after a “night out on the town.” ANZ J Surg. (2006) 76:110–2. 10.1111/j.1445-2197.2006.03664.x 16626342

[59] Uchal M Tjugum J Martinsen E Qiu X Bergamaschi R. The impact of sleep deprivation on product quality and procedure effectiveness in a laparoscopic physical simulator: a randomized controlled trial. Am J Surg. (2005) 189:753–7. 10.1016/j.amjsurg.2005.03.021 15910732

[60] Elizabeth E Michael M David H Erie J Effect of sleep deprivation on the performance of simulated anterior segment surgical skill. Can J Ophthalmol. (2011) 46:61–5. 10.3129/i10-112 21283160

[61] Lehmann KS Martus P Little-Elk S Maass H Holmer C Zurbuchen U. Impact of sleep deprivation on medium-term psychomotor and cognitive performance of surgeons: prospective cross-over study with a virtual surgery simulator and psychometric tests. Surgery. (2010) 147:246–54. 10.1016/j.surg.2009.08.007 20109624

[62] Olasky J Chellali A Sankaranarayanan G Zhang L Miller A De S. Effects of sleep hours and fatigue on performance in laparoscopic surgery simulators. Surg Endosc Other Interv Tech. (2014) 28:2564–8. 10.1007/s00464-014-3503-0 24671352 PMC4126861

[63] Schlosser K Maschuw K Kupietz E Weyers P Schneider R Rothmund M Call-associated acute fatigue in surgical residents-subjective perception or objective fact? A cross-sectional observational study to examine the influence of fatigue on surgical performance. World J Surg. (2012) 36:2276–87. 10.1007/s00268-012-1699-5 22752051

[64] Veddeng A Husby T Engelsen IB Kent A Flaatten H. Impact of night shifts on laparoscopic skills and cognitive function among gynecologists. Acta Obstet Gynecol Scand. (2014) 93:1255–61. 10.1111/aogs.12496 25178160

[65] Yi WS Hafiz S Sava JA. Effects of night-float and 24-h call on resident psychomotor performance. J Surg Res. (2013) 184:49–53. 10.1016/j.jss.2013.03.029 23587456

[66] Jakubowicz DM Price EM Glassman HJ Gallagher AJ Mandava N Ralph WP. Effects of a twenty-four hour call period on resident performance during simulated endoscopic sinus surgery in an accreditation council for graduate medical education-compliant training program. Laryngoscope. (2005) 115:143–6. 10.1097/01.mlg.0000150689.77764.ad 15630383

[67] Lehmann KS Ritz JP Maass H Çakmak HK Kuehnapfel UG Germer CT Bretthauer G Buhr H.J. A prospective randomized study to test the transfer of basic psychomotor skills from virtual reality to physical reality in a comparable training setting. Ann Surg. (2005) 241:442–9. 10.1097/01.sla.0000154552.89886.91 15729066 PMC1356982

[68] Tomasko JM Pauli EM Kunselman AR Haluck RS. Sleep deprivation increases cognitive workload during simulated surgical tasks. Am J Surg. (2012) 203:37–43. 10.1016/j.amjsurg.2011.08.009 22079034

[69] Sahayadhas A Sundaraj K Murugappan M. Detecting driver drowsiness based on sensors: a review. Sensors. (2012) 12:16937–53. 10.3390/s121216937 23223151 PMC3571819

[70] Lynn Caldwell J Chandler JF Hartzler BM Battling fatigue in aviation: recent advancements in research and practice. J Med Sci. (2012) 32:47–56. Available online at: https://apps.dtic.mil/dtic/tr/fulltext/u2/a561167.pdf

[71] Young MS Steel T Rail worker fatigue: identification, management and countermeasures. Proc Inst Mech Eng Part F J Rail Rapid Transit. (2017) 231:1098–106. 10.1177/0954409716675383

[72] Berry RB Brooks R Gamaldo CE Harding SM Lloyd RM Marcus CL The AASM Manual for the Scoring of Sleep and Associated Events: Rules, Terminology and Technical Specifications, Version 2.2. Darien, IL: American Academy of Sleep Medicine (2015). Available online at: https://aasm.org/resources/pdf/scoring-manual-preface.pdf

[73] Sauvet F Bougard C Coroenne M Lely L Van Beers P Elbaz M. In-flight automatic detection of vigilance states using a single EEG channel. IEEE Trans Biomed Eng. (2014) 61:2840–7. 10.1109/TBME.2014.2331189 24967979

[74] Garcés Correa A Orosco L Laciar E. Automatic detection of drowsiness in EEG records based on multimodal analysis. Med Eng Phys. (2014) 36:244–9. 10.1016/j.medengphy.2013.07.011 23972332

[75] Hajinoroozi M Mao Z Jung TP Lin CT Huang Y EEG-based prediction of driver's cognitive performance by deep convolutional neural network. Signal Process Image Commun. (2016) 47:549–55. 10.1016/j.image.2016.05.018

[76] Johnson RR Popovic DP Olmstead RE Stikic M Levendowski DJ Berka C. Drowsiness/alertness algorithm development and validation using synchronized EEG and cognitive performance to individualize a generalized model. Biol Psychol. (2011) 87:241–50. 10.1016/j.biopsycho.2011.03.003 21419826 PMC3155983

[77] Berka C Levendowski DJ Lumicao MN Yau A Davis G Zivkovic VT. EEG correlates of task engagement and mental workload in vigilance, learning, and memory tasks. Aviat Space Environ Med. (2007) 78:B231–44. 17547324

[78] Berka C Izzetoglu K Bunce S Onaral B Pourrezaei K Chance B Real-time analysis of EEG indexes of alertness, cognition, and memory acquired with a wireless EEG headset. Int J Hum Comput Interact. (2004) 17:211–27. 10.1207/s15327590ijhc1702_3

[79] Dinges DF Mallis MM Maislin G Powell JW Evaluation of Techniques for Ocular Measurement as an Index of Fatigue and as the Basis for Alertness Management. Washington, DC: National Highway Traffic Safety Administration (1998). Available online at: https://trid.trb.org/view.aspx?id=647942

[80] Zhang Z Zhang J A new real-time eye tracking based on nonlinear unscented Kalman filter for monitoring driver fatigue. J Control Theory Appl. (2010) 8:181–8. 10.1007/s11768-010-8043-0

[81] Liu D Sun P Xiao YQ Yin Y Drowsiness detection based on eyelid movement. In: 2010 Second International Workshop on Education Technology and Computer Science. Wuhan (2010). p. 49–52. 10.1109/ETCS.2010.292

[82] Yin B-C Fan X Sun Y-F Multiscale dynamic features based driver fatigue detection. Int J Pattern Recognit Artif Intell. (2009) 23:575–89. 10.1142/S021800140900720X

[83] Vural E Cetin M Ercil A Littleworth G Bartlett M Movellan J Drowsy driver detection through facial movement analysis. Human Computer Interact. (2007) 4796:6–18. 10.1007/978-3-540-75773-3_2

[84] Marzano C Fratello F Moroni F Pellicciari MC Curcio G Ferrara M. Slow eye movements and subjective estimates of sleepiness predict EEG power changes during sleep deprivation. Sleep. (2007) 30:610–6. 10.1093/sleep/30.5.610 17552376

[85] Abe T Nonomura T Komada Y Asaoka S Sasai T Ueno A. Detecting deteriorated vigilance using percentage of eyelid closure time during behavioral maintenance of wakefulness tests. Int J Psychophysiol. (2011) 82:269–74. 10.1016/j.ijpsycho.2011.09.012 21978525

[86] Amirian I Andersen LT Rosenberg J Gögenur I. Working night shifts affects surgeons' biological rhythm. Am J Surg. (2014) 210:389–95. 10.1016/j.amjsurg.2014.09.035 25678472

[87] Chua EC-P Tan W-Q Yeo S-C Lau P Lee I Mien IH. Heart rate variability can be used to estimate sleepiness-related decrements in psychomotor vigilance during total sleep deprivation. Sleep. (2012) 35:325–34. 10.5665/sleep.1688 22379238 PMC3274333

[88] Ancoli-Israel S Cole R Alessi C Chambers M Moorcroft W Pollak CP. The role of actigraphy in the study of sleep and circadian rhythms. Sleep. (2003) 26:342–92. 10.1093/sleep/26.3.342 12749557

[89] McCormick F Kadzielski J Landrigan CP Evans B Herndon JH Rubash HE. Surgeon fatigue. Arch Surg. (2012) 147:430–5. 10.1001/archsurg.2012.84 22785637

[90] Hursh SR Balkin TJ Miller JC Eddy DR The fatigue avoidance scheduling tool: Modeling to minimize the effects of fatigue on cognitive performance. SAE Trans. (2004) 113:111–9. 10.4271/2004-01-2151

[91] Younes M Soiferman M Thompson W Giannouli E. Performance of a new portable wireless sleep monitor. J Clin Sleep Med. (2017) 13:245–58. 10.5664/jcsm.6456 27784419 PMC5263080

[92] Nakamura T Alqurashi YD Morrell MJ Mandic D. Hearables: automatic overnight sleep monitoring with standardised in-ear EEG sensor. IEEE Trans Biomed Eng. (2019). 10.1109/TBME.2019.2911423 31021747

[93] Driskell JE Mullen B. The efficacy of naps as a fatigue countermeasure: a meta-analytic integration. Hum Factors. (2005) 47:360–77. 10.1518/0018720054679498 16170944

[94] Lovato N Lack L. The effects of napping on cognitive functioning. Prog Brain Res. (2010) 185:155–66. 10.1016/B978-0-444-53702-7.00009-9 21075238

[95] Jewett ME Wyatt JK Ritz-De Cecco A Khalsa SB Dijk DJ Czeisler CA. Time course of sleep inertia dissipation in human performance and alertness. J Sleep Res. (1999) 8:1–8. 10.1111/j.1365-2869.1999.00128.x 10188130

[96] Achermann P Werth E Dijk DJ Borbely AA. Time course of sleep inertia after nighttime and daytime sleep episodes. Arch Ital Biol. (1995) 134:109–19. 8919196

[97] Ferrara M De Gennaro L. The sleep inertia phenomenon during the sleep-wake transition: theoretical and operational issues. Aviat Space Environ Med. (2000) 71:843–8. 10954363

[98] Ferrara M De Gennaro L Bertini M. Time-course of sleep inertia upon awakening from nighttime sleep with different sleep homeostasis conditions. Aviat Sp Environ Med. (2000) 71:225–9. 10716166

[99] Tempesta D Cipolli C Desideri G De Gennaro L Ferrara M. Can taking a nap during a night shift counteract the impairment of executive skills in residents? Med Educ. (2013) 47:1013–21. 10.1111/medu.12256 24016171

[100] Milner CE Cote KA. Benefits of napping in healthy adults: Rmpact of nap length, time of day, age, and experience with napping. J Sleep Res. (2009) 18:272–81. 10.1111/j.1365-2869.2008.00718.x 19645971

[101] Richardson GS Carskadon MA Orav EJ Dement WC. Circadian variation of sleep tendency in elderly and young adult subjects. Sleep. (1982) 5(Suppl. 2):S82–94. 10.1093/sleep/5.S2.S82 7156658

[102] Phipps-Nelson J Redman JR Dijk D-J Rajaratnam SMW. Daytime exposure to bright light, as compared to dim light, decreases sleepiness and improves psychomotor vigilance performance. Sleep. (2003) 26:695–700. 10.1093/sleep/26.6.695 14572122

[103] Zhou J Liu D Li X Ma J Zhang J Fang J. Pink noise: effect on complexity synchronization of brain activity and sleep consolidation. J Theor Biol. (2012) 306:68–72. 10.1016/j.jtbi.2012.04.006 22726808

[104] Clark I Landolt HP. Coffee, caffeine, and sleep: a systematic review of epidemiological studies and randomized controlled trials. Sleep Med Rev. (2016) 31:70–8. 10.1016/j.smrv.2016.01.006 26899133

[105] Landolt HP Rétey JV Tönz K Gottselig JH Khatami R Buckelmüller I. Caffeine attenuates waking and sleep electroencephalographic markers of sleep homeostasis in humans. Neuropsychopharmacology. (2004) 29:1933–9. 10.1038/sj.npp.1300526 15257305

[106] Einöther SJL Giesbrecht T. Caffeine as an attention enhancer: reviewing existing assumptions. Psychopharmacology. (2013) 225:251–74. 10.1007/s00213-012-2917-4 23241646

[107] Nawrot P Jordan S Eastwood J Rotstein J Hugenholtz A Feeley M. Effects of caffeine on human health. Food Addit Contam. (2003) 20:1–30. 10.1080/0265203021000007840 12519715

[108] Humayun MU. Quantitative measurement of the effects of caffeine and propranolol on surgeon hand tremor. Arch Ophthalmol. (1997) 115:371. 10.1001/archopht.1997.01100150373010 9076210

[109] Fargen KM Turner RD Spiotta AM. Factors that affect physiologic tremor and dexterity during surgery: a primer for neurosurgeons. World Neurosurg. (2015) 86:384–9. 10.1016/j.wneu.2015.10.098 26585718

[110] Wood S Sage JR Shuman T Anagnostaras SG. Psychostimulants and cognition: a continuum of behavioral and cognitive activation. Pharmacol Rev. (2014) 66:193–221. 10.1124/pr.112.007054 24344115 PMC3880463

[111] Yerkes RM Dodson JD The relation of strength of stimulus to rapidity of habit-formation. J Comp Neurol Psychol. (1908) 18:459–82. 10.1002/cne.920180503

[112] Retey JV Adam M Honegger E Khatami R Luhmann UFO Jung HH. A functional genetic variation of adenosine deaminase affects the duration and intensity of deep sleep in humans. Proc Natl Acad Sci USA. (2005) 102:15676–81. 10.1073/pnas.0505414102 16221767 PMC1266101

[113] Åkerstedt T Gillberg M. Sleep duration and the power spectral density of the EEG. Electroencephalogr Clin Neurophysiol. (1986) 64:119–22. 10.1016/0013-4694(86)90106-9 2424728

[114] Borbély AA Baumann F Brandeis D Strauch I Lehmann D. Sleep deprivation: Effect on sleep stages and EEG power density in man. Electroencephalogr Clin Neurophysiol. (1981) 51:483–93. 10.1016/0013-4694(81)90225-X 6165548

[115] Retey JV Adam M Gottselig JM Khatami R Durr R Achermann P. Adenosinergic mechanisms contribute to individual differences in sleep deprivation-induced changes in neurobehavioral function and brain rhythmic activity. J Neurosci. (2006) 26:10472–9. 10.1523/JNEUROSCI.1538-06.2006 17035531 PMC6674679

[116] Aggarwal R Mishra A Crochet P Sirimanna P Darzi A. Effect of caffeine and taurine on simulated laparoscopy performed following sleep deprivation. Br J Surg. (2011) 98:1666–72. 10.1002/bjs.7600 21761394

[117] Czeisler CA Walsh JK Roth T Hughes RJ Wright KP Kingsbury L. Modafinil for excessive sleepiness associated with shift-work sleep disorder. N Engl J Med. (2005) 353:476–86. 10.1056/NEJMoa041292 16079371

[118] Billiard M Narcolepsy: current treatment options and future approaches. Neuropsychiatr Dis Treat. (2008) 4:557–66. 10.2147/NDT.S484 18830438 PMC2526380

[119] Sugden C Housden CR Aggarwal R Sahakian BJ Darzi A. Effect of pharmacological enhancement on the cognitive and clinical psychomotor performance of sleep-deprived doctors. Ann Surg. (2012) 255:222–7. 10.1097/SLA.0b013e3182306c99 21997802

[120] Klerman EB Beckett SA Landrigan CP. Applying mathematical models to predict resident physician performance and alertness on traditional and novel work schedules. BMC Med Educ. (2016) 16:239. 10.1186/s12909-016-0751-9 27623842 PMC5022151

[121] Jensen A Milner R Fisher C Gaughan J Rolandelli R Grewal H Short-term sleep deficits do not adversely affect acquisition of laparoscopic skills in a laboratory setting. Surg Endosc Other Interv Tech. (2004) 18:948–53. 10.1007/s00464-003-8225-7 15095080

[122] Goel N Neurobehavioral effects and biomarkers of sleep loss in healthy adults. Curr Neurol Neurosci Rep. (2017) 17:89 10.1007/s11910-017-0799-x 28944399

[123] Lucassen EA Piaggi P Dsurney J de Jonge L Zhao XC Mattingly MS. Sleep extension improves neurocognitive functions in chronically sleep-deprived obese individuals. PLoS ONE. (2014) 9:1–9. 10.1371/journal.pone.0084832 24482677 PMC3903365

[124] Goel N Rao H Durmer J Dinges D. Neurocognitive consequences of sleep deprivation. Semin Neurol. (2009) 29:320–39. 10.1055/s-0029-1237117 19742409 PMC3564638

[125] Abe T Mollicone D Basner M Dinges DF. Sleepiness and safety: where biology needs technology. Sleep Biol Rhythms. (2014) 12:74–84. 10.1111/sbr.12067 24955033 PMC4061704

[126] Kosmadopoulos A Sargent C Zhou X Darwent D Matthews RW Dawson D. The efficacy of objective and subjective predictors of driving performance during sleep restriction and circadian misalignment. Accid Anal Prev. (2017) 99:445–51. 10.1016/j.aap.2015.10.014 26534845

[127] Helmreich RL. On error management: lessons from aviation. BMJ. (2000) 320:781–5. 10.1136/bmj.320.7237.781 10720367 PMC1117774

[128] Ramakrishnan S Wesensten NJ Kamimori GH Moon JE Balkin TJ Reifman J. A unified model of performance for predicting the effects of sleep and caffeine. Sleep. (2016) 39:1827–41. 10.5665/sleep.6164 27397562 PMC5020365

[129] Vital-Lopez FG Ramakrishnan S Doty TJ Balkin TJ Reifman J. Caffeine dosing strategies to optimize alertness during sleep loss. J. Sleep Res. (2018) 27:e12711. 10.1111/jsr.12711 29808510

[130] Van Dongen HPA Baynard MD Maislin G Dinges DF. Systematic interindividual differences in neurobehavioral impairment from sleep loss: evidence of trait-like differential vulnerability. Sleep. (2004) 27:423–33. 15164894

[131] Goel N Dinges DF. Predicting risk in space: genetic markers for differential vulnerability to sleep restriction. Acta Astronaut. (2012) 77:207–13. 10.1016/j.actaastro.2012.04.002 23524958 PMC3602842

[132] Kuna ST Maislin G Pack FM Staley B Hachadoorian R Coccaro EF. Heritability of performance deficit accumulation during acute sleep deprivation in twins. Sleep. (2012) 35:1223–33. 10.5665/sleep.2074 22942500 PMC3413799

[133] Goel N Dinges DF. Behavioral and genetic markers of sleepiness. J Clin Sleep Med. (2011) 7:11–13. 10.5664/jcsm.1348 22003324 PMC3190416

[134] Viola AU Archer SN James LM Groeger JA Lo JCY Skene DJ. PER3 polymorphism predicts sleep structure and waking performance. Curr Biol. (2007) 17:613–8. 10.1016/j.cub.2007.01.073 17346965

[135] Mignot E Young T Lin L Finn L. Nocturnal sleep and daytime sleepiness in normal subjects with HLA-DQB1^*^0602. Sleep. (1999) 22:347–52. 10341385

[136] Sturm L Dawson D Vaughan R Hewett P Hill AG Graham JC. Effects of fatigue on surgeon performance and surgical outcomes: a systematic review. ANZ J Surg. (2011) 81:502–9. 10.1111/j.1445-2197.2010.05642.x 22295360

[137] Walker MP Brakefield T Seidman J Morgan A Hobson JA Stickgold R. Sleep and the time course of motor skill learning. Learn Mem. (2003) 10:275–84. 10.1101/lm.58503 12888546 PMC202318

[138] Walker MP Stickgold R Alsop D Gaab N Schlaug G. Sleep-dependent motor memory plasticity in the human brain. Neuroscience. (2005) 133:911–7. 10.1016/j.neuroscience.2005.04.007 15964485

[139] Peigneux P Laureys S Delbeuck X Maquet P. Sleeping brain, learning brain. The role of sleep for memory systems. Neuroreport. (2001) 12:A111–24. 10.1097/00001756-200112210-00001 11742260

[140] Gulati T Guo L Ramanathan DS Bodepudi A Ganguly K. Neural reactivations during sleep determine network credit assignment. Nat Neurosci. (2017) 20:1277–84. 10.1038/nn.4601 28692062 PMC5808917

[141] Curcio G Ferrara M De Gennaro L. Sleep loss, learning capacity and academic performance. Sleep Med Rev. (2006) 10:323–37. 10.1016/j.smrv.2005.11.001 16564189

[142] Aggarwal R Grantcharov T Moorthy K Hance J Darzi A. A competency-based virtual reality training curriculum for the acquisition of laparoscopic psychomotor skill. Am J Surg. (2006) 191:128–33. 10.1016/j.amjsurg.2005.10.014 16399123

[143] Ahmed K Van Poppel H Khan MS Van Cleynenbreugel B Artibani W Rischmann P. Development of a standardised training curriculum for robotic surgery: a consensus statement from an international multidisciplinary group of experts. BJU Int. (2014) 116:93–101. 10.1111/bju.12974 25359658

[144] Van Dongen KW Tournoij E Van Der Zee DC Schijven MP Broeders IAMJ. Construct validity of the LapSim: can the LapSim virtual reality simulator distinguish between novices and experts? Surg Endosc Other Interv Tech. (2007) 21:1413–7. 10.1007/s00464-006-9188-2 17294307

[145] Selvander M Åsman P. Cataract surgeons outperform medical students in Eyesi virtual reality cataract surgery: evidence for construct validity. Acta Ophthalmol. (2013) 91:469–74. 10.1111/j.1755-3768.2012.02440.x 22676143

[146] Bergqvist J Person A Vestergaard A Grauslund J. Establishment of a validated training programme on the Eyesi cataract simulator. A prospective randomized study. Acta Ophthalmol. (2014) 92:629–34. 10.1111/aos.12383 24612448

[147] Ramakrishnan S Lu W Laxminarayan S Wesensten NJ Rupp TL Balkin TJ. Can a mathematical model predict an individual's trait-like response to both total and partial sleep loss? J Sleep Res. (2015) 24:262–9. 10.1111/jsr.12272 25559055

[148] Liu J Ramakrishnan S Laxminarayan S Balkin TJ Reifman J. Real-time individualization of the unified model of performance. J Sleep Res. (2017) 26:820–31 10.1111/jsr.12535 28436072

[149] Lim J Dinges DF. Sleep deprivation and vigilant attention. Ann NY Acad Sci. (2008) 1129:305–22. 10.1196/annals.1417.002 18591490

[150] Chee MWL Goh CSF Namburi P Parimal S Seidl KN Kastner S. Effects of sleep deprivation on cortical activation during directed attention in the absence and presence of visual stimuli. Neuroimage. (2011) 58:595–604. 10.1016/j.neuroimage.2011.06.058 21745579 PMC3159770

[151] Chee MW Tan JC Zheng H Parimal S Weissman DH Zagorodnov V. Lapsing during sleep deprivation is associated with distributed changes in brain activation. J Neurosci. (2008) 28:5519–28. 10.1523/JNEUROSCI.0733-08.2008 18495886 PMC6670628

[152] Finelli LA Baumann H Borbély AA Achermann P. Dual electroencephalogram markers of human sleep homeostasis: correlation between theta activity in waking and slow-wave activity in sleep. Neuroscience. (2000) 101:523–9. 10.1016/S0306-4522(00)00409-7 11113301

[153] De Gennaro L Marzano C Veniero D Moroni F Fratello F Curcio G. Neurophysiological correlates of sleepiness: a combined TMS and EEG study. Neuroimage. (2007) 36:1277–87. 10.1016/j.neuroimage.2007.04.013 17524675

[154] Siclari F Tononi G. Local aspects of sleep and wakefulness. Curr Opin Neurobiol. (2017) 44:222–7. 10.1016/j.conb.2017.05.008 28575720 PMC6445546

[155] Nir Y Andrillon T Marmelshtein A Suthana N Cirelli C Tononi G. Selective neuronal lapses precede human cognitive lapses following sleep deprivation. Nat Med. (2017) 23:1474–80. 10.1038/nm.4433 29106402 PMC5720899

[156] Ayalon RD Friedman F. The effect of sleep deprivation on fine motor coordination in obstetrics and gynecology residents. Am J Obstet Gynecol. (2008) 199:576.e1–e5. 10.1016/j.ajog.2008.06.080 18822404

[157] Reznick RK Folse JR. Effect of sleep deprivation on the performance of surgical residents. Am J Surg. (1987) 154:520–5. 10.1016/0002-9610(87)90269-8 3674301

[158] Hirkani MA Yogi J Effect of sleep deprivation on finger dexterity in resident doctors. Natl J Physiol Pharm Pharmacol. (2017) 7:697–700. 10.5455/njppp.2017.7.0204102032017

[159] Deaconson T O'Hair D Marlon L Lee M Schueneman A Condon R Sleep deprivation and residents performance. JAMA (1988) 260:1721–7. 10.1001/jama.1988.03410120067029 3411755

[160] Escobar-Castillejos D Noguez J Neri L Magana A Benes B. A review of simulators with haptic devices for medical training. J Med Syst. (2016) 40:1–22. 10.1007/s10916-016-0459-8 26888655

[161] Harders M. VR-based simulators for training in minimally invasive. IEEE Comput Graph Appl. (2007) 27:54–66. 10.1109/MCG.2007.51 17388203

